# When suffering becomes a game: a critical examination of gamification in medical education and its implications for compassion and empathy

**DOI:** 10.3389/fmed.2026.1810141

**Published:** 2026-04-01

**Authors:** Changiz Mohiyeddini

**Affiliations:** Oakland University William Beaumont School of Medicine, Rochester, MI, United States

**Keywords:** compassion, empathy decline, gamification, hidden curriculum, medical education, objectification, patient-centered care

## Abstract

Gamification has rapidly proliferated across medical education, promising enhanced engagement, improved knowledge retention, and more motivated learners. Yet the uncritical embrace of game mechanics in contexts defined by human suffering raises profound questions that the field has largely failed to confront. This critical essay first proposes a typology distinguishing gamification, serious games, and game-based learning to identify which specific design elements pose the greatest ethical risks in which learning contexts. It then examines the philosophical and empirical tensions inherent in translating patient pain, vulnerability, and existential distress into point systems, leaderboards, and competitive challenges. Drawing on Nussbaum’s objectification framework, Buber’s I-Thou philosophy, evidence from systematic reviews, and the well-documented phenomenon of empathy decline during medical training, this essay argues that competitive, rewards-driven gamification, when applied to clinical scenarios without structured reflection, risks accelerating the very dehumanization it sometimes claims to counteract. The essay acknowledges that cooperative, narrative-driven, and perspective-taking game designs can support empathy development, and proposes concrete ethical guidelines for educators seeking to balance innovation with humanistic values.

## Introduction: the seductive promise and the unasked question

The integration of gamification into medical education has accelerated with remarkable momentum. A bibliometric analysis by Yıldı et al. (1) documented that publications on gamification in health education surged dramatically after 2018, with the highest output in 2023, spanning domains from nursing to critical care to evidence-based medicine. The enthusiasm is understandable. Traditional medical education, reliant on didactic lectures and passive absorption, often fails to sustain student engagement or produce deep learning. Gamification, with its promise of interactivity, immediate feedback, and motivational scaffolding, appears to offer a compelling remedy.

Yet in the rush to gamify, a fundamental question has gone largely unasked: What happens to our relationship with human suffering when we translate it into the language of games? When a patient’s cancer diagnosis becomes a clinical reasoning card game, when managing a psychiatric crisis becomes a competitive simulation scored on a leaderboard, when the agony of chronic pain becomes a data point in a progress bar–what cognitive and moral transformations are we silently engineering in the minds of future physicians?

This essay does not argue that gamification has no place in medical education. There are contexts–anatomy quizzes, pharmacology drills, procedural skills training–where game elements may legitimately enhance learning without ethical compromise. Nor does it argue that all game-based approaches are ethically equivalent; as the typology below demonstrates, the ethical risk varies dramatically with design choices. Rather, this essay contends that the field has adopted gamification with a troubling absence of critical reflection about its implications for the affective and relational dimensions of medical practice, particularly compassion and empathy toward patients whose suffering constitutes the very substrate being “played.”

## A necessary typology: not all games are created equal

A significant limitation of the current literature–and one that this revised analysis seeks to address–is the tendency to treat gamification as a monolithic construct. In practice, game-based approaches in medical education span a wide spectrum, and their ethical implications differ substantially depending on design choices. Following Watsjold et al. (2), three distinct categories can be identified.

First, gamification in the strict sense refers to the layering of game elements–points, badges, leaderboards, progress bars, and competitive rankings–onto existing educational activities that would function without them. A pharmacology quiz that award points for speed and accuracy, or a clinical reasoning module that ranks students on a leaderboard, exemplifies this approach. The learning activity exists independently; the game layer provides extrinsic motivational scaffolding. It is this category that poses the greatest ethical risk when applied to patient-centered clinical content, because the competitive and rewards-driven mechanics operate as an overlay on material that involves human suffering, without requiring the learner to engage with that suffering as such.

Second, serious games unify learning objectives and game objectives such that gameplay cycles themselves produce learning outcomes. Examples include GridlockED, a board game in which success requires understanding patient flow through an emergency department, and Night Shift, a branching-narrative video game designed to improve trauma triage decisions (2). The critical distinction is that in a well-designed serious game, the game mechanics can be aligned with rather than imposed upon the learning objectives. When the game mechanic is cooperation rather than competition, or when the narrative structure centers the patient’s perspective rather than the student’s score, serious games can potentially support relational learning.

Third, game-based learning encompasses the broader use of play, role-play, and interactive simulation in education. This includes cooperative escape rooms, patient-perspective narrative games, empathy board games that require learners to adopt the patient’s role, and virtual reality experiences that place students in the phenomenological position of the patient (3, 4). These approaches may incorporate game elements but are defined by their pedagogical orientation rather than their rewards structure.

This typology matters because the ethical concerns raised in this essay apply with varying force across the spectrum. The critique is most urgent for competitive, rewards-driven gamification applied to clinical scenarios involving patient suffering–particularly when such gamification occurs without structured debriefing or reflective integration. It applies with less force to cooperative serious games designed with empathy as an explicit learning objective, and with least force to narrative and perspective-taking game designs that position the learner as witness rather than competitor. The failure to make these distinctions has allowed the field to deflect critique of specific harmful designs by pointing to the successes of fundamentally different approaches.

## The empirical landscape: what we know and what we have not bothered to measure

The evidence base for gamification in medical education is simultaneously expansive and strikingly shallow. A systematic review by van Gaalen et al. (5) identified 44 empirical studies evaluating gamification in health professions education and found that no negative outcomes were reported–a finding that should provoke skepticism rather than comfort. The absence of negative findings likely reflects not the absence of harm but the absence of measurement. When studies focus exclusively on knowledge scores, engagement metrics, and satisfaction surveys, they are structurally incapable of detecting erosion in compassion, empathy, or moral sensitivity.

A revealing analysis by Huang et al. (6), applying the Structure of Observed Learning Outcomes (SOLO) taxonomy to gamified medical education, found that 78% of reviewed studies operated at the lowest cognitive level–uni-structural recall of isolated facts. Only a single study (4%) reached the highest level, where empathy was even considered as a learning outcome. The authors noted that this study merely suggested gamification could improve students’ “intention to convey empathy,” a far cry from demonstrating that gamification fosters genuine empathic understanding of another’s suffering. The distinction between performing empathy and experiencing it is not trivial; it is, arguably, the distinction that defines the difference between a technically competent clinician and a healer.

The broader gamification-in-healthcare literature echoes this concern. A 2025 systematic review on gamification in digital mental health interventions identified what the authors termed an “Engagement–Efficacy–Ethics Trilemma,” revealing that increased app usage does not consistently translate to better therapeutic outcomes and that persuasive design introduces risks of manipulation and psychological distress (7). A recent scoping review of gamification for clinical reasoning found that serious games were the most common technique (45.3%), followed by escape rooms (11.3%) and board/card games (7.5%), yet the heterogeneity in design, duration, and complexity made it difficult to compare results, underscoring the need for standardized reporting frameworks and a clearer taxonomy of gamification techniques (8). The tension between engagement and genuine benefit is not merely a technical problem–it is a moral one.

## The philosophical problem: objectification, dehumanization, and the threshold between them

A central philosophical challenge for this analysis is to draw a clear boundary between the clinically necessary objectification that is inherent in medical practice and the ethically problematic dehumanization that this essay warns against. These are not identical phenomena, and conflating them weakens the critique. The distinction can be sharpened by drawing on Nussbaum (9) influential analysis of objectification, which identifies seven dimensions: instrumentality (treating a person as a tool), denial of autonomy, inertness (treating as lacking agency), fungibility (treating as interchangeable), violability, ownership, and denial of subjectivity. Not all forms of objectification are morally equivalent, and not all are avoidable in clinical practice.

In medicine, some degree of objectification is not only unavoidable but necessary. As Svenaeus (10) argued, when a patient presents with stomach pain, they expect the physician to objectify their body–to order blood tests, imaging, and physical examination. These procedures are not alienating or dehumanizing in themselves; they become so only when the healthcare professional fails to simultaneously approach the patient as a worried and suffering person. The critical threshold, following Svenaeus’s phenomenological analysis, is whether objectification occurs within a relational context that preserves the patient’s subjectivity, or whether it occurs in a manner that renders subjectivity structurally invisible.

This distinction illuminates precisely where different gamification designs fall on the ethical spectrum. Consider a competitive clinical reasoning game that rewards diagnostic speed with points and ranks students on a leaderboard. Three of Nussbaum’s dimensions are activated simultaneously: the patient scenario becomes instrumental (a means to earning points), fungible (interchangeable with any other scenario of equivalent difficulty), and denied subjectivity (the patient’s inner world is irrelevant to success). When these dimensions operate together, especially under conditions of time pressure and social comparison, the threshold from clinically necessary objectification to ethically problematic dehumanization is crossed–not because the student consciously dehumanizes, but because the game’s design grammar makes the patient’s personhood structurally irrelevant to the learning activity.

Martin Buber’s distinction between the I-Thou and I-It modes of relating provides the relational framework for this analysis. In the I-Thou encounter, the other is met as a whole being–irreducible, singular, and commanding of genuine presence. In the I-It mode, the other becomes an object to be categorized and instrumentalized. Buber considered the therapist-patient relationship a special form of I-Thou relation, sustained by what he called “inclusion”: the clinician’s capacity to experience the patient’s suffering from both their own and the patient’s standpoint simultaneously.

Gamification that relies on competitive mechanics, by its very design grammar, promotes an I-It orientation. When a patient scenario is rendered as a “challenge” to be “completed,” the patient ceases to be a Thou and becomes an It–a variable in an optimization problem. However–and this is a crucial qualification–game designs that center narrative, cooperation, and perspective-taking can potentially support the I-Thou orientation. A cooperative serious game in which students must work together to understand a patient’s illness experience, or a narrative game that places the learner in the patient’s phenomenological position, operates on fundamentally different relational principles than a competitive leaderboard. The ethical evaluation must therefore be specific to design, not categorical against all game-based approaches.

Pellegrino and Thomasma’s philosophy of medicine, which situates the healing relationship at the center of clinical practice, further illuminates the danger of unreflective gamification. They argue that the dominant unspoken philosophy of American medicine is a Cartesian reductionism that treats the body as a machine and the physician as a technician. Competitive gamification, far from challenging this framework, perfects it. It provides the most sophisticated behavioral engineering yet devised to train future clinicians in the habits of reductionism, all while wrapping the training in the dopaminergic pleasures of gameplay.

## The empathy crisis: gamification as accelerant

The well-documented decline in medical student empathy during training provides essential context for evaluating gamification’s likely effects. A systematic review by Neumann et al. (11) found that three longitudinal and six cross-sectional studies demonstrated significant decreases in empathy during medical school, with the steepest decline occurring during the transition to clinical training. Hojat et al. (12), in a nationwide cross-sectional study of 10,751 students at DO-granting medical schools, confirmed this pattern. A subsequent systematic review and thematic synthesis by Howick et al. (13) identified remarkably consistent themes explaining empathy decline: the hidden curriculum, stressful working environments, poor role modeling, and cynicism bred by the gap between what is formally taught about empathy and what is implicitly rewarded in clinical practice.

The hidden curriculum is particularly relevant here. As Howick et al. (14) outlined in their roadmap for an empathic hidden curriculum, the implicit messages medical students receive include that biomedical knowledge trumps relational competence, that emotional engagement with patients is inefficient and professionally risky, and that clinical detachment is a sign of maturity rather than a failure of moral development. Competitive gamification, when applied to clinical scenarios, risks becoming a particularly potent vector for these hidden messages. When the “game” rewards speed, accuracy, and competitive ranking, it implicitly communicates that the patient encounter is something to be won rather than something to be entered. When empathy is absent from the scoring rubric–as it is in the overwhelming majority of gamified medical education interventions–students learn that empathy is irrelevant to excellence.

Moreover, competitive gamification introduces specific psychological risks. A randomized cross-over study by Kirsch and Spreckelsen (15) warned that high competition can correlate with feelings of inferiority, shame, rising stress, anxiety, and even depression among medical students. Importantly, these risks are associated specifically with competitive mechanics–leaderboards, rankings, and win/lose conditions–rather than with game-based learning *per se*. In environments where competitive gamification intersects with the already-elevated stress of medical training, the conditions for empathy erosion are not merely preserved–they are intensified. The student who “loses” the clinical simulation game does not simply fail a test; they internalize that the patient’s suffering was the arena of their failure, subtly poisoning the affective well from which compassion must be drawn.

## The trivialization problem: Can we play with suffering?

A recurrent concern in the literature–acknowledged but insufficiently explored–is that gamification risks trivializing complex health issues. Castellano-Tejedor and Cencerrado (16) noted that “ensuring the simplicity of the games does not trivialize complex health issues” remains a significant challenge. A study on ethical and pedagogical reflections around serious games in patient education directly posed the question: Can we play with our health? The French research team found that while both patients and caregivers expressed favorable stances toward gamification, the ethical permissibility of the practice depended on caregivers serving as “tutors and debriefers”–a safeguard that is frequently absent in self-directed gamified learning platforms (17).

The trivialization problem operates at multiple levels. At the surface level, there is the aesthetic incongruity of rendering a patient’s existential crisis in the visual and sonic language of games–progress bars, achievement badges, celebratory sound effects. At a deeper level, gamification enforces a narrative structure that is fundamentally incompatible with the phenomenology of suffering. Games have clear objectives, defined endpoints, and measurable success criteria. Suffering does not. A patient with treatment-resistant depression, a family receiving a terminal diagnosis, a person living with chronic pain that defies explanation–these experiences resist the tidy narrative arc that gamification demands. When medical education forces suffering into game structures, it does not make suffering more understandable; it makes it invisible in its most essential dimensions.

There is also an equity dimension to the trivialization problem. As Timmermans and Almeling (18) argued regarding the commodification of suffering in health systems, the translation of human distress into quantifiable, manageable units serves institutional efficiency but often obscures the structural determinants of illness and the lived experience of marginalized populations. Gamification, which inherently requires the standardization and quantification of outcomes, risks replicating these patterns within the educational setting, teaching future clinicians to see suffering through the lens of metrics rather than meaning.

## The motivation paradox: extrinsic rewards and the erosion of moral purpose

Self-determination theory, frequently invoked to justify gamification, actually provides one of the strongest theoretical bases for concern. The theory distinguishes between extrinsic motivation–driven by external rewards, competition, and social comparison–and intrinsic motivation, which arises from genuine interest, personal meaning, and alignment with one’s values. The overwhelming majority of gamified medical education interventions rely on extrinsic motivators: points, leaderboards, badges, and competitive rankings. A Delphi study by Wang et al. (19) identified the top gamification elements as integration with instructional objectives, game rules, rapid feedback, fairness, and points/scoring. Notably absent from the list of key elements: compassion, empathy, or moral formation.

Decades of research in motivational psychology have demonstrated that extrinsic rewards can undermine intrinsic motivation–a phenomenon known as the overjustification effect. When medical students are extrinsically rewarded for engaging with patient scenarios, there is a genuine risk that the intrinsic moral motivation that drew them to medicine–the desire to alleviate suffering, the call to serve the vulnerable, the recognition of shared humanity–is gradually displaced by the drive to accumulate points. The student who entered medical school wanting to help becomes the student who wants to win. This concern applies most directly to competitive gamification designs; cooperative and narrative-driven approaches, by contrast, may support autonomy and relatedness–two of the three basic needs identified by self-determination theory–when they invite learners to engage meaningfully with the patient’s perspective rather than compete against peers.

## When games can heal: acknowledging the positive potential of game-based learning for empathy

Intellectual honesty and the strength of the critique both require acknowledging that some game-based approaches have demonstrated genuine potential for supporting empathy and perspective-taking in health professions education. This acknowledgment does not weaken the argument; rather, it sharpens it by focusing the critique on the specific design elements most likely to produce harm.

Several examples illustrate that game design, when oriented toward relational learning rather than competition, can operate on fundamentally different ethical principles. Hudnall et al. (20) developed The Empathy Project, a cooperative, facilitator-led card game designed for palliative care education in which learners practice responding to patients’ emotional expressions. The game produced statistically significant growth in learners’ ability to name emotions, reflect understanding, and explore emotional content–precisely the empathic skills that competitive gamification neglects. Critically, the game’s effectiveness depended on the presence of a communication expert as facilitator, confirming that game mechanics alone are insufficient without relational scaffolding.

Similarly, Lu et al. (3) developed an empathy board game for nursing education in Taiwan that required learners to simulate various patient roles and develop diverse interpersonal dialogues. Through cooperative game elements–storytelling, real-time responses, peer learning, and role-reversal–students who played patient roles were able to perceive different levels of comfort and develop the cognitive, emotional, and expressive dimensions of empathy. More recently, Mohan et al. (4) used a role-playing photographic game based on a nurse-cancer patient interaction that produced significant improvements in empathy scores 7 days after the intervention. Virtual reality experiences that place learners in the patient’s phenomenological position–experiencing what it feels like to lose cognitive function, to navigate a hospital while disoriented, to receive devastating news–have similarly shown promise for enhancing affective empathy through embodied perspective-taking (21).

What distinguishes these approaches from the competitive gamification that is the focus of this critique? Three design principles emerge. First, the relational orientation is cooperative rather than competitive: learners work together or adopt the patient’s role, rather than competing against each other for points. Second, the narrative is centered on the patient’s experience rather than on the learner’s performance metrics. Third, structured debriefing or facilitation is embedded in the design, ensuring that the game experience is reflectively integrated rather than consumed as entertainment. When these principles are present, game-based learning can function as a vehicle for I-Thou encounter rather than an engine of I-It habituation.

## Toward an ethical framework: concrete guidelines for gamification in clinical education

Drawing together the typological, philosophical, and empirical analyses presented above, the following ethical framework is proposed for educators considering gamification in clinical medical education. These guidelines are intended to be specific and actionable, recognizing that the question is not whether to use game elements but which elements, in which contexts, with which safeguards.

First, educators should apply the typological distinction as a threshold question: Does the proposed intervention layer competitive game mechanics onto clinical content (gamification), or does it integrate learning objectives with game mechanics in a manner that centers the patient’s experience (serious game or game-based learning)? The former requires heightened ethical scrutiny; the latter may proceed with standard educational evaluation, provided empathy outcomes are measured.

Second, competitive elements–leaderboards, rankings, win/lose conditions, time-pressured scoring–should not be applied to scenarios in which patient suffering constitutes the educational content. These mechanics activate the Nussbaum dimensions of instrumentality, fungibility, and denial of subjectivity simultaneously, and the evidence from Kirsch and Spreckelsen (15) suggests they introduce psychological risks that compound empathy decline. Competitive mechanics remain appropriate for contexts where the “patient” is an anatomical model, a pharmacological dataset, or a procedural skill–domains where objectification is clinically necessary and where no patient subjectivity is at stake.

Third, all gamified clinical education should include structured debriefing that explicitly reconnects the game experience to the humanity of the patient. The evidence from Coelho and Reis (17) and Hudnall et al. (20) consistently indicates that game mechanics without facilitator-guided reflection fail to produce relational learning. Debriefing should specifically address: what the patient in the scenario might have been experiencing, what aspects of the patient’s personhood the game made visible or invisible, and how the student’s emotional response during gameplay connects to the affective demands of clinical practice.

Fourth, outcome evaluations of gamified curricula must include validated measures of empathy, compassion, and relational competence–not merely knowledge retention and engagement metrics. The near-total absence of empathy measurement in the gamification literature (6) is not a minor methodological gap; it is a structural blind spot that permits harm to go undetected.

Fifth, when game-based approaches are used for clinical communication and patient-centered care objectives, cooperative and narrative-driven designs should be preferred over competitive formats. Designs that position the learner in the patient’s role, that require collaborative problem-solving, or that center patient narratives can support the I-Thou orientation that clinical practice demands.

## Conclusion: the moral stakes of how we teach

Medical education is not merely the transmission of knowledge and skills; it is the formation of moral agents who will hold the suffering of others in their hands. Every pedagogical choice sends implicit messages about what matters, what counts, and how the patient should be regarded. When we gamify clinical education without critical reflection on which game mechanics are being deployed and in which relational contexts, we risk communicating that suffering is a problem to be solved rather than an experience to be witnessed, that patients are variables rather than persons, and that the physician’s primary relationship is not with the patient but with the scoreboard.

The field urgently needs to move beyond the undifferentiated question of whether gamification “works”–measured narrowly as knowledge retention and engagement–to the more precise question of which gamification designs, applied in which contexts, produce which effects on empathy, compassion, and relational competence. This requires the typological clarity to distinguish competitive from cooperative designs, the philosophical rigor to draw boundaries between clinically necessary objectification and ethically problematic dehumanization, and the empirical humility to measure what we have so far neglected.

Francis Peabody, Dean of Harvard Medical School, observed over a century ago that the depersonalization of hospital care was the predictable consequence of institutional culture rather than individual moral failure. The same insight applies today. If we build educational systems that structurally exclude empathy from their rewards mechanisms, we should not be surprised when the physicians who emerge from those systems struggle to offer it. The patient’s suffering is not a game. But some games, carefully designed, can teach us to be more present to that suffering. Our responsibility as educators is to know the difference.

## Data Availability

The original contributions presented in this study are included in this article/supplementary material, further inquiries can be directed to the corresponding author.
